# An Unexpected Finding of Poorly Differentiated Thyroid Carcinoma in a Toxic Thyroid Nodule

**DOI:** 10.1210/jcemcr/luad052

**Published:** 2023-05-23

**Authors:** Kimberly Yuang, Huda Al-Bahadili, Alan Chang

**Affiliations:** Department of Medicine, Division of Endocrinology, Diabetes, and Metabolism, Stony Brook University Hospital, Stony Brook, NY 11794, USA; Department of Medicine, Division of Endocrinology, Diabetes, and Metabolism, Stony Brook University Hospital, Stony Brook, NY 11794, USA; Department of Medicine, Division of Endocrinology, Diabetes, and Metabolism, Stony Brook University Hospital, Stony Brook, NY 11794, USA

**Keywords:** toxic thyroid nodule, poorly differentiated thyroid carcinoma, hyperthyroidism

## Abstract

Poorly differentiated thyroid carcinoma (PDTC) is a rare entity of thyroid cancer with an intermediate clinical behavior between differentiated and anaplastic thyroid cancer. Here we present a patient who was referred to the endocrinology clinic for evaluation of hyperthyroidism and multinodular goiter. Due to presence of right toxic thyroid nodules and compressive symptoms, the patient underwent right lobectomy and isthmectomy, where surgical pathology revealed PDTC in the right thyroid lobe. Based on this unusual case of malignancy within a toxic nodule, we propose further evaluation of hot nodules with concerning features such as growth rate. Furthermore, exploration of relative sodium iodine symporter (NIS) expression in PDTC may help us better understand how iodine uptake changes as PDTC develops, which may impact our approach to assessing and treating PDTC in the future.

## Introduction

Poorly differentiated thyroid carcinoma (PDTC) accounts for about 6% of thyroid cancers and is thought to have an intermediate clinical behavior between differentiated and anaplastic thyroid cancer [1]. Although described earlier, PDTC was introduced to the World Health Organization (WHO) in 2004. Currently, the Turin proposal is accepted by the WHO as the uniform diagnostic criteria of PDTC [1]. According to the Turin proposal, PDTC is defined histologically as having a solid, trabecular, insular growth pattern with lack of papillary thyroid cancer nuclear features, and one of the following: convoluted nuclei, mitotic index of at least 3 per 10 high power fields (HPF), and tumor necrosis [1].

PDTC tend to present late clinically [2]. Thus, accurate diagnosis and timely recognition of its distinct features are important. Here we present a male patient with hyperthyroidism, right toxic thyroid nodules, negative fine needle aspiration (FNA), and PDTC on surgical pathology. In addition, we reviewed the literature to explore the accuracy of FNAs and characteristics of PDTC that may result in a positive iodine uptake scan.

## Case Presentation

A 66-year-old male with a urothelial carcinoma and history of metastatic transitional cell carcinoma to the lung, with a history of left upper lobectomy, presented to the endocrinology clinic for evaluation and management of hyperthyroidism and multinodular goiter. Upon evaluation, he endorsed right-sided neck discomfort, but denied dysphagia, odynophagia, voice changes, heat intolerance, palpitations, tremors, diarrhea, unexplained anxiety, or weight loss. There was no history of neck irradiation or family history of thyroid cancer. On physical examination, he was found to have an asymmetric thyroid gland with right lobe enlargement, firm and nontender. He had no proptosis, exophthalmos, lid lag, or fine tremors. Six months prior to his endocrinology office visit, the patient was evaluated by otolaryngology (ear/nose/throat; ENT) for an enlarged right thyroid lobe with calcifications seen on computed tomography (CT) of the chest without intravenous contrast and diffuse hypermetabolic activity in right thyroid lobe on positron emission tomography (PET) CT. An in-office thyroid ultrasound was performed, followed by ultrasound-guided FNA of the right isoechoic solid thyroid nodule. It was unclear why the right hypoechoic solid thyroid nodule was not biopsied at that time. The patient was concerned about palpable right thyroid lobe enlargement and right lobectomy was discussed but not pursued immediately. A thyroid function test was performed, revealing a suppressed thyroid-stimulating hormone (TSH) level with elevated free thyroxine (T4) which prompted referral to the endocrinology office. When taking the previous findings into consideration, along with the presence of hyperthyroidism, the decision was made to obtain an iodine-123 (I-123) thyroid uptake scan in addition to checking thyroid-stimulating immunoglobulins and TSH receptor antibodies to rule out Graves disease.

## Diagnostic Assessment

**Figure 1.**
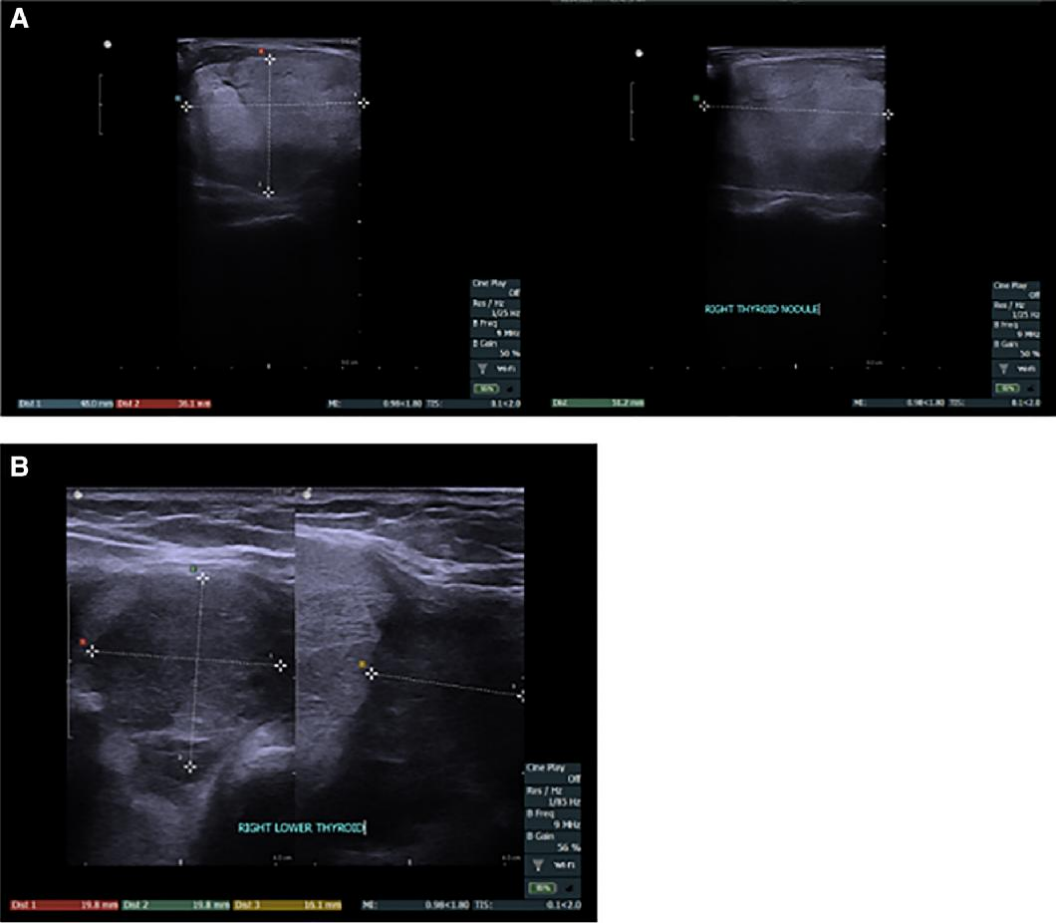
Thyroid ultrasound. A, Right superior-middle pole solid isoechoic thyroid nodule (4.8 × 3.6 × 5.1 cm). B, Right lower solid hypoechoic thyroid nodule (2.0 × 2.0 × 1.6 cm).

**Figure 2.**
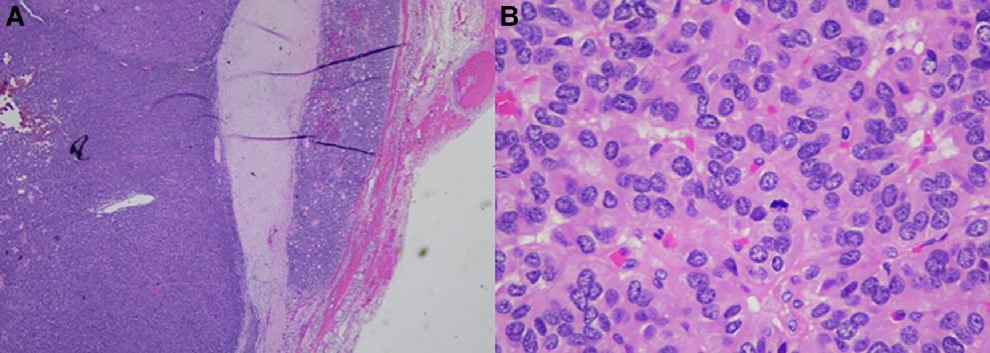
A, Capsular invasion and extracapsular nodule. B, Mitotic figure in the center.

**Figure 3.**
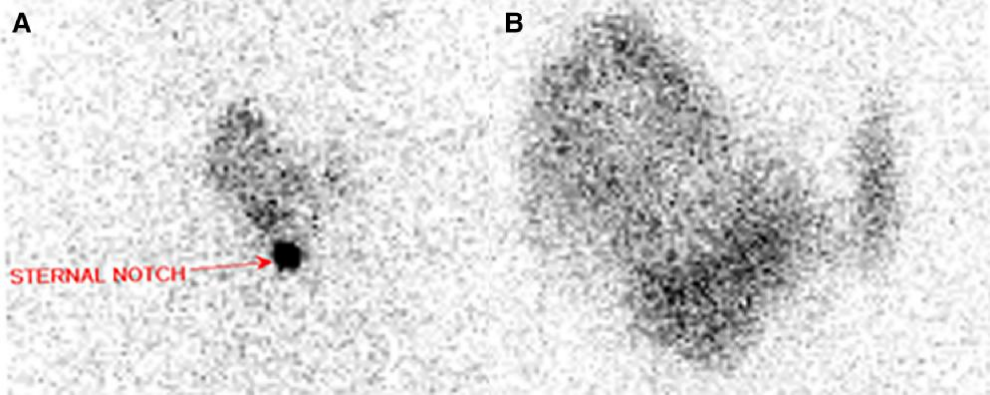
I-123 uptake and scan. A, I-123 scan of the thyroid relative to the sternal notch. B, Closer look at the iodine uptake within the right thyroid lobe.

The thyroid ultrasound performed in the ENT office prior to the endocrinology office visit (shown in Fig. 1), revealed 2 distinct right thyroid nodules: an isoechoic solid nodule measuring 4.8 × 3.6 × 5.1 cm, encompassing most of the right lobe; and a hypoechoic solid lower pole nodule measuring 2.0 × 2.0 × 1.6 cm with no suspicious lymphadenopathy. FNA of the right isoechoic solid thyroid nodule demonstrated an abundant amount of follicular epithelium with predominantly bland follicles seen individually and in sheet-like aggregates, ample colloid, and scattered macrophages, consistent with Bethesda II category (shown in Fig. 2). Thyroid function tests showed a serum TSH of 0.163 μIU/mL (0.163 mIU/L) (normal range, 0.270-4.200 μIU/mL [0.270-4.200 mIU/L]), and free T4 was 1.92 ng/dL (24.71 pmol/L) (normal range, 0.93-1.70 ng/dL [12.00-21.93 pmol/L]). Thyroid-stimulating immunoglobulins and TSH receptor antibodies were negative. Following laboratory workup, an I-123 thyroid uptake scan revealed 5% uptake at 6 hours (normal, 5%-15%) and 6% uptake at 24 hours (normal 15%-35%) (shown in Fig. 3) with a very heterogeneous radiotracer activity consistent with a large right thyroid nodule.

## Treatment

In view of the compressive symptoms and enlarged right thyroid lobe, the patient underwent right lobectomy and isthmectomy.

## Outcome and Follow-up

**Figure 4.**
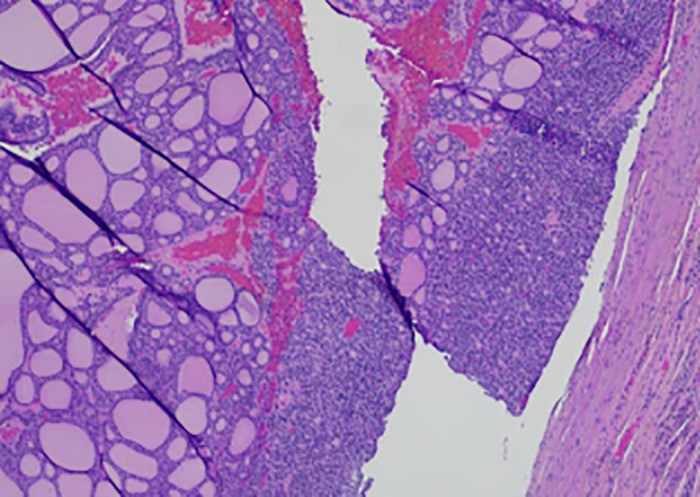
Surgical pathology showed solid/trabecular growth pattern with well-differentiated component of follicular carcinoma.

Surgical pathology (shown in Fig. 4) showed a unifocal PDTC with lymphatic invasion, measuring 6.5 cm in the largest dimension, positive for TTF-1, PAX8, thyroglobulin, and negative for GATA3. The margins were negative for tumor. Given the large tumor size and aggressive histology, the patient's condition would typically warrant further treatment with completion left lobectomy and radioactive iodine ablation, or potentially, external beam radiation without completion. However, in the context of his recurrent urothelial carcinoma, the patient was managed with active surveillance while undergoing further therapy for his urothelial carcinoma. A few weeks after right lobectomy and isthmectomy, the patient's thyroid function test showed hypothyroidism with serum TSH 7.28 μIU/mL (7.28 mIU/L) (normal, 0.270-4.200 μIU/mL [0.270-4.200 mIU/L]), free T4 0.68 ng/dL (8.75 pmol/L) (normal, 0.93-1.70 ng/dL [12.00-21.93 pmol/L]). For postsurgical hypothyroidism, the patient was started on levothyroxine 88 mcg every morning.

## Discussion

Our patient's case presents multiple interesting findings. First, the presence of discordance in results of the negative FNA and the surgical pathology results showing PDTC. FNA is considered the gold standard for diagnosis of thyroid nodules >1 cm with suspicious features [3]. There are, however, false negatives, with one retrospective study reporting a 10.5% false-negative rate, and a sensitivity of 89.5%, specificity of 98%, false positive rate of 1.9%, and accuracy of 97% [4]. While some authors who propose that false-negative FNAs may be related to nodule size, the American Thyroid Association (ATA) notes that the data are unclear since some of the studies demonstrate selection bias, where only a subset of participants undergoes preoperative FNA, and possible sampling error due to palpation rather than ultrasound-guided FNA procedure [3].

As PDTC has significantly lower 5, 10, and 15 year-survival rates than well-differentiated thyroid cancers [2], the impact of false-negative FNA results on mortality would likely be more significant in patients with PDTC. In the retrospective analysis by Kim et al, 6 cases of PDTC treated between January 2015 and November 2018 at Gachon University Gil Medical Center were examined, all with FNAs that were not consistent with Bethesda criteria V (malignancy) [5]. According to Bethesda criteria, 2 were III, 2 were IV, 1 was V, and 1 was II [5]. It was postulated that these findings were in part due to the complexity of obtaining the sample that result in pauci-cellularity, cystic fluid, blood, drying artifact, or calcified material affecting the sample, leading to difficulty in correct FNA diagnosis [5]. It was also noted that the mitosis and necrosis frequently seen on histology of PDTC are uncommon among FNA samples due to the presence of only small foci of PDTC in many cases [5]. While there are established adequacy criteria to help reduce some of the inaccuracies related to obtaining the sample, continued monitoring of nodules for concerning features such as accelerated growth [6] and consideration of the appropriateness of surgical intervention based upon the clinical picture in addition to FNA results may benefit patients.

Another interesting feature of our case presentation was the I-123 uptake pattern demonstrated. It is generally accepted that thyroid nodules that have avid uptake of I-123 (“hot nodules”) are less likely to harbor a malignancy compared with a nodule that is not iodine-avid. Some clinicians think of PDTC as thyroid cancer that does not trap iodine on radioiodine scans and mention that the ATA recommends CT/PET scans for initial staging and monitoring of patients with PDTC [7]. Although rare, there have been documented hot nodules that harbor thyroid cancers [8].

PDTC is thought to arise from either de-differentiation of a well-differentiated thyroid cancer or a de novo mutation of benign thyroid follicular cells with both early and late driver mutations [9]. In a publication by Patel et al, it was noted that PDTC has the potential to concentrate iodine as they arise from follicular epithelium. This potential serves as a basis of postoperative radioactive iodine therapy [2].

The exact mechanism of iodine uptake in a patient with PDTC is unclear, although the variation in ability to uptake iodine might be related to variation in sodium iodine symporter (NIS) expression in different subtypes of PDTC. Nikitski et al suggested that there may be unique subtypes of PDTC with variation of NIS expression [10]. In this study, the authors looked at mouse models using a STRN-ALk gene fusion as early driver mutation, and p53 loss as a late driver mutation [10]. After introducing these mutations, the researchers found that 26 mice had well-differentiated thyroid cancer, 21 had PDTC meeting Turin criteria, and 8 had anaplastic thyroid cancer [10]. Upon histological analysis of the PDTC tumors, 2 subtypes were discovered, differentiated by the nucleus to cytoplasm ratio and immunoreactivity to thyroglobulin and E-cadherin [10]. The authors noted that NIS was decreased in both subtypes, but significantly more so in subtype 2 [10]. Nikitski et al also observed that PDTC subtype 1 may possibly precede PDTC subtype 2 based on ultrasound images used to reconstruct the nodules’ growth and the presence of both subtypes within the same thyroid [10]. The relative presence of more NIS in one subtype could potentially mean greater uptake on the iodine uptake scan. Nonetheless, in vivo models to examine radioactive iodine uptake and concentration are still needed to further explore treatment and/or surveillance implications in patients with toxic nodules and PDTC [10].

## Learning Points

- We recommend consideration of nodule growth in determining treatment/intervention rather than FNA alone.
- Examination of NIS expression may lend keen insights into the variation in iodine uptake among different subtypes/stages of PDTC, and this should be further explored in humans [10].

## Contributors

All authors made individual contributions to authorship. A.C. was involved in the diagnosis and management of this patient. A.C., H.A., and K.Y. were involved in manuscript writing and submission. All authors reviewed and approved the final draft.

## Data Availability

Data sharing is not applicable to this article as no datasets were generated or analyzed during the current study.
